# 443. Pre-vaccination Antibody Titers Against Seasonal Coronaviruses And Antibody Responses to the Pfizer-BioNTech BNT162b2 COVID-19 mRNA Vaccine in Healthcare Workers

**DOI:** 10.1093/ofid/ofab466.642

**Published:** 2021-12-04

**Authors:** Eric Laing, Si’Ana Coggins, Kevin Schully, Emily Samuels, Emilie Goguet, Matthew Moser, Belinda Jackson-Thompson, Simon Pollett, David Tribble, Julian Davies, Luca Illinik, Monique Hollis-Perry, Santina Maiolatesi, Christopher Duplessis, Kathleen Ramsey, Anatalio Reyes, Yolanda Alcorta, Mimi Wong, Orlando Ortega, Gregory Wang, Edward Parmelee, Alyssa Lindrose, Timothy Burgess, Christopher C Broder, Edward Mitre

**Affiliations:** 1 Uniformed Services University of the Health Sciences, Bethesda, Maryland; 2 HJF, USUHS, Bethesda, Maryland; 3 BDRD NMRC, Frederick, Maryland; 4 CTC, NMRC, Silver Spring, Maryland; 5 HJF, CTC NMRC, Silver Spring, Maryland; 6 CTC, NMRC, General Dynamics Information Technology, Silver Spring, Maryland; 7 Infectious Disease Clinical Research Program, Bethesda, Maryland; 8 USUHS, Bethesda, Maryland

## Abstract

**Background:**

The Prospective Assessment of SARS-CoV-2 Seroconversion (PASS) study is following over 200 healthcare workers who have received the Pfizer-BioNTech BNT162b2 COVID-19 mRNA vaccine. A major aim of the study is to determine whether baseline antibody titers against the seasonal human coronaviruses are associated with altered levels of vaccine-induced antibody responses to SARS-CoV-2.

**Methods:**

Serial serum samples obtained pre-vaccination and 1 month after the second dose were tested for IgG antibodies against the full pre-fusion spike protein and the receptor binding domain (RBD) of SARS-CoV-2, as well as the full pre-fusion spike proteins of OC43, HKU1, 229E, and NL63. Antibodies were measured using highly sensitive and specific multiplex assays based on Luminex-xMAP technology.

**Results:**

Preliminary analyses of the first 103 subjects in whom we have 1 month post-vaccination serum demonstrate development of high IgG geometric mean titers (GMT) to both the full spike protein (GMT: 13,685, 12,014-15,589, 95% CI) and the RBD (GMT: 19,448, 17,264-21,908, 95% CI) of SARS-CoV-2 after the 2^nd^ vaccine dose. Preliminary analysis demonstrates no association between baseline antibody titers against spike protein of OC43 and antibody titers against SARS-CoV-2 spike protein (Pearson’s r-value= 0.13, *P*-value= 0.21) or RBD (Pearson’s r-value= 0.09, *P*-value= 0.36) one month after vaccination. Future analyses will evaluate whether there is an association with baseline seasonal coronavirus antibody titers and either SARS-CoV-2 neutralization titers or anti-SARS-CoV-2 spike protein titers at 6 months after vaccination.

**Conclusion:**

These preliminary results suggest that baseline antibody responses to seasonal coronaviruses neither boost nor impede SARS-CoV-2 vaccine-induced antibody responses. Longitudinal sampling will enable assessment of vaccine durability and determination of whether baseline seasonal coronavirus antibody levels are associated with altered duration of detectable COVID-19 vaccine-induced antibody responses.

**Disclosures:**

**Simon Pollett, MBBS**, **Astra Zeneca** (Other Financial or Material Support, HJF, in support of USU IDCRP, funded under a CRADA to augment the conduct of an unrelated Phase III COVID-19 vaccine trial sponsored by AstraZeneca as part of USG response (unrelated work)) **David Tribble, M.D., DrPH**, **Astra Zeneca** (Other Financial or Material Support, HJF, in support of USU IDCRP, funded under a CRADA to augment the conduct of an unrelated Phase III COVID-19 vaccine trial sponsored by AstraZeneca as part of USG response (unrelated work))

